# Few-Shot Object Detection in Remote Sensing Images via Data Clearing and Stationary Meta-Learning

**DOI:** 10.3390/s24123882

**Published:** 2024-06-15

**Authors:** Zijiu Yang, Wenbin Guan, Luyang Xiao, Honggang Chen

**Affiliations:** College of Electronics and Information Engineering, Sichuan University, Chengdu 610065, China; yangzijiu@stu.scu.edu.cn (Z.Y.); 2021222050079@stu.scu.edu.cn (W.G.); xiaoluyang@stu.scu.edu.cn (L.X.)

**Keywords:** few-shot object detection, remote sensing images, meta-learning, incompletely annotated objects

## Abstract

Nowadays, the focus on few-shot object detection (FSOD) is fueled by limited remote sensing data availability. In view of various challenges posed by remote sensing images (RSIs) and FSOD, we propose a meta-learning-based Balanced Few-Shot Object Detector (B-FSDet), built upon YOLOv9 (GELAN-C version). Firstly, addressing the problem of incompletely annotated objects that potentially breaks the balance of the few-shot principle, we propose a straightforward yet efficient data clearing strategy, which ensures balanced input of each category. Additionally, considering the significant variance fluctuations in output feature vectors from the support set that lead to reduced effectiveness in accurately representing object information for each class, we propose a stationary feature extraction module and corresponding stationary and fast prediction method, forming a stationary meta-learning mode. In the end, in consideration of the issue of minimal inter-class differences in RSIs, we propose inter-class discrimination support loss based on the stationary meta-learning mode to ensure the information provided for each class from the support set is balanced and easier to distinguish. Our proposed detector’s performance is evaluated on the DIOR and NWPU VHR-10.v2 datasets, and comparative analysis with state-of-the-art detectors reveals promising performance.

## 1. Introduction

In recent decades, advancements in remote sensing technology have led to the development of spaceborne sensors, which now offer sub-meter spatial resolution, comparable to airborne images from a few decades ago [1]. Operating continuously, these sensors have generated pretty valuable data, making automatic image interpretation and object detection increasingly essential.

With the advancement of convolutional neural networks (CNNs), significant breakthroughs have been achieved in the field of object detection, with many high-performance detectors demonstrating outstanding performance under extensive training data [2,3,4]. However, in practical applications, the labeled data obtained from remote sensing sensors are limited, and acquiring them is costly and difficult. CNN-based detectors are prone to overfitting and exhibiting poor performance when faced with such limited data. To address this issue, many studies have begun researching how to train detectors that can perform well with a limited amount of data, giving rise to few-shot object detection (FSOD) [5,6,7].

Numerous FSOD methods adhere to a two-stage training approach, which entails an initial base training phase using an established base dataset containing ample training samples to acquire general prior knowledge. Subsequently, the base-trained detector undergoes fine-tuning using a few-shot dataset comprising target categories. The categories within the base dataset are referred to as base classes, while the target categories added in the few-shot dataset are denoted as novel classes. Currently, there are two mainstream approaches to addressing FSOD: meta-learning and transfer learning. They both follow a two-stage training paradigm, the effectiveness of which has been demonstrated across various scenarios [8,9]. Specifically, meta-learning-based methods stand out for their ability to rapidly adapt to extremely limited sample scenarios and exhibit strong generalization capabilities [10,11,12], which makes them an excellent choice for situations where training data are scarce. Nonetheless, several challenges exist when dealing with remote sensing images (RSIs) in FSOD.

**Figure 1:**
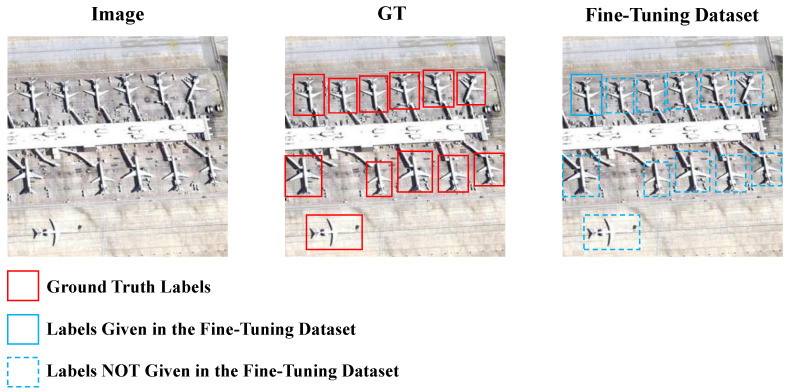
Schematic diagram illustrating the issue of IAO problem in the fine-tuning dataset. During training, only a subset of labels is utilized for supervision, while the remaining labels, due to the constraints of the N-way-K-shot principle, cannot be provided, resulting in confusion for the detector.

Firstly, during the phase of fine-tuning on the novel class, the fine-tuning dataset needs to be constructed according to the N-way-K-shot principle [13]. For instance, when N = 10 and K = 3, the fine-tuning dataset should consist of 10 categories, with each category containing three labels. However, the correspondence between the three labels and the images is not strictly one-to-one. In existing remote sensing datasets, it is challenging to ensure one label corresponds to one image, as a single image usually contains multiple objects. To adhere to the N-way-K-shot principle, only a portion of these labels from these images can be utilized as training inputs. Consequently, incomplete labeling frequently occurs in the fine-tuning dataset. As shown in Figure 1, when fine-tuning for the “airplane” class, as a novel class, only certain objects in the image are annotated. The given labels provide positive guidance to the detector, whereas missing labels may lead the detector to regard the objects as background, causing significant confusion, referred to as the incompletely annotated objects (IAO) issue. Current FSOD methods addressing the IAO issue [14,15] employ pseudo-labels or novel classifiers to mitigate the problem. However, these approaches rely on the model’s current understanding of novel class knowledge, which is evidently much lower than that of base classes in few-shot conditions. Since the root cause of the IAO issue lies in data processing problems, we believe that addressing it from the perspective of data is straightforward and effective.

**Figure 2:**
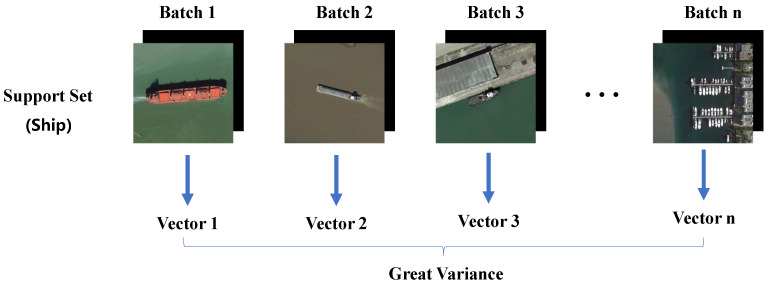
Schematic diagram of support set feature extraction structure in FSODM [11] and LMFSODet [17]. Suppose one of the classes in the support set is “ship”.

Additionally, a significant concern within meta-learning frameworks is the method of integrating support set features with query set features. Presently, two predominant aggregation approaches are recognized: class-specific aggregation (CSA) [12] and class-agnostic aggregation (CAA) [16]. CSA [12] merges features of the same class from both the support set and the query set, enhancing the detector’s ability to memorize specific objects. Conversely, CAA [16] allows for the fusion of object features from different classes between the support set and the query set, thereby aiding the detector in distinguishing between classes. Moreover, there exists a technique for encoding support set features into vector form and assisting the query set through channel-wise multiplication, which offers operational simplicity, lightweight parameters, and universality. Li et al. [11] employed this method in RSIs, where support set images and their corresponding label mask images were jointly fed into convolutional layers, ultimately encoding feature vectors to assist the query set features. However, Han et al. [16] suggested that such vectors may be influenced by data scarcity and variations in examples, failing to adequately represent the entire class distribution. This limitation can be partly attributed to [11] introducing background information during encoding. Guan et al. [17] proposed encoding only the objects, which to some extent reduces the variance. However, objects in RSIs exhibit significant intra-class differences, and encoding only the objects may still result in large variance in the output vectors, as shown in Figure 2. Hence, we hold the view that during training, enabling the support set’s features to be derived not solely from a specific set of data but rather by synthesizing the auxiliary features of the support set would help stabilize the output vectors of the support set and enhance their auxiliary effects.

**Figure 3:**
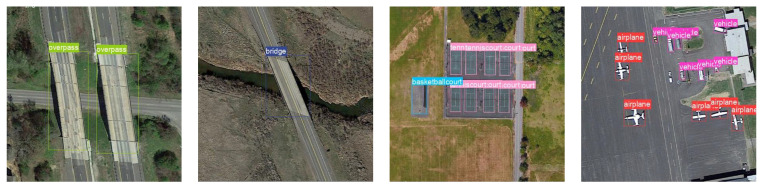
Visualization of some categories of objects from the DIOR dataset.

In the end, objects within RSIs obtained from remote sensing sensors often exhibit minor inter-class differences (MIDs), manifested in various aspects such as color and shape, as shown in Figure 3. Relying solely on support set features to assist the detection does not effectively enhance the classifier’s classification ability. The prevailing method for addressing the MID issue is contrastive learning. However, these approaches [18,19] significantly increase model complexity and are challenging to directly apply to meta-learning-based few-shot object detectors. We fully exploit the distinct characteristics of various objects in the support set. By enhancing the detector’s ability to distinguish between different components of the support set’s output vectors through cross-entropy, its knowledge of distinguishing different classes can be reinforced.

To address the aforementioned challenges and considering the limited computational resources in practical applications that require models to be as lightweight as possible, we propose a novel Balanced Few-Shot Object Detector based on the single-stage detector YOLOv9 (GELAN-C version) [20]. Given its consideration of balance and stability in handling samples for FSOD across all its designed components, it is fittingly dubbed B-FSDet. To begin with, in an effort to achieve a genuine balanced input sample during fine-tuning, we propose a straightforward yet highly effective data clearing strategy (DCS). The DCS operates on fine-tuning dataset images, removing redundant objects based on the complete set of labels and a subset of labels used for few-shot learning. Notably, this process is lightweight as it does not rely on complex deep-learning-based image inpainting techniques. Instead, it simply employs white Gaussian noise (WGN) to replace the objects. Importantly, our detector’s loss computation only involves valuable positive samples, thus minimizing the impact of substituted WGN on the detector performance. What is more, to ensure that the output vectors from the support set comprehensively represent the features of each class, we introduce the stationary feature extraction module (SFEM), based on [11,20]. We also apply dynamic exponential moving average (DEMA) to the output vectors to mitigate the impact stemming from unstable model parameters during training and the addition of novel classes during fine-tuning. In the meantime, we propose a stationary and fast prediction method (SFPM) coupled with SFEM that does not rely on support set images specifically matched with the object with high detecting speed. Instead, it randomly selects from the support set class library, demonstrating significant robustness and efficiency. Finally, we propose the inter-class discrimination support loss (ICDSL). Building upon the existing detection head, we augment the detection head with the decoding function for support set vectors. ICDSL is calculated between the decoded results and the ground truth class provided by the support set to strengthen the detection head’s ability to discriminate different classes.

In summary, the main contributions of this paper can be summarized as follows:

- We propose a novel Balanced Few-Shot Object Detector (B-FSDet), based on the YOLOv9 (GELAN-C version) [20] and meta-learning. Considering the limited computational resources, B-FSDet achieves remarkably high detection accuracy with a low parameter count, and effectively addresses numerous challenges prevalent in RSIs.
- To ensure genuine balance in input samples during fine-tuning, we introduce DCS, which removes redundant objects from fine-tuning dataset images. The lightweight process employs WGN to replace the redundant objects, resulting in precise alignment of objects with labels and adherence to the N-way-K-shot principle.
- To make the output vectors comprehensively represent the features of each class in the support set, we introduce SFEM and SFPM. The two parts construct a stationary meta-learning mode, improving the robustness of the detector.
- Addressing the issue of minor inter-class differences, we propose ICDSL to strengthen the detection head’s ability to discriminate between classes.

## 2. Related Work

### 2.1. Object Detection in Remote Sensing Images

General object detectors can be divided into two main types: two-stage detectors and one-stage detectors. Two-stage detectors, like Faster R-CNN [4], start by using a region proposal network to suggest potential foreground regions. Then, they extract fixed-size features for each proposal through ROI pooling and use a module for transformation, along with a classifier and a regressor predicting final bounding boxes. On the other hand, one-stage detectors, such as the YOLO series [3,20,21,22], simultaneously predict bounding boxes and class scores based on predefined anchor boxes, resulting in very fast inference speeds and high accuracy.

Numerous detectors have achieved commendable performance in addressing object detection tasks in natural scene images. However, RSIs differ from natural scene images in that they are captured from a bird’s-eye-view perspective, introducing challenges such as complex backgrounds, diverse scales of foreground objects, and significant variability in object orientations. Massive studies have been launched to tackle these problems. To tackle the challenge of rotation invariance in feature learning, Mei et al. [23] introduced a groundbreaking approach known as cyclic polar coordinate convolutional layer. Recognizing the complex nature of aerial images, which often feature large sensory areas and diverse scales of objects, Deng et al. [24] proposed a multiscale object proposal network comprising three branches dedicated to predicting multiscale proposals. Given the inadequacy of horizontal bounding boxes in accurately representing aerial object shapes, numerous studies like [25,26] have shifted their focus to detecting novel objects using rotated bounding boxes. In a related vein, Lian et al. [27] developed an innovative contextual background attack framework aimed at deceiving aerial detectors. Hui et al. [28] considered the tiny size of objects in RSIs and proposed SEB-YOLO network to fully catch the targets. Additionally, extensive research such as [27,29] has been devoted to the advancement of object detection in RSIs.

While the aforementioned detectors have shown promising results in object detection in RSIs, their performance heavily relies on the availability of extensive annotated data. In practical scenarios, especially in the remote sensing domain, collecting large amounts of labeled data is labor-intensive. As a result, these detectors experience a significant decrease in performance when dealing with limited-sample scenarios.

### 2.2. Few-Shot Object Detection in Remote Sensing Images

Due to the difficulty in acquiring large amounts of annotated data, FSOD has emerged as a popular research area. One of the current mainstream FSOD methods is meta-learning. Meta-learning typically involves training a meta-learner on a large dataset containing various tasks or domains, where each task consists of a few-shot learning scenario. During meta-training, the meta-learner learns to generalize across tasks and acquires knowledge about how to effectively adapt to new tasks given limited training data. Classic meta-learning-based methods include Meta-RCNN [12] and FSODM [11], both of which utilize reweighting vectors to assist query set feature aggregation. Additionally, VFA [16] was the pioneer in integrating variational feature learning into FSOD to enhance feature robustness. Building upon this, Lu et al. [30] introduced the information-coupled prototype elaboration method to generate more distinct and representative prototypes for individual query images. Their approach has demonstrated improved performance in FSOD tasks. Nonetheless, the output features of the support set generated by these methods often exhibit high variance, making the assistance to the query set unstable. Hence, more efforts should be launched to make the assistance from the support set effective and comprehensive. Additionally, Li et al. [31] proposed a class margin equilibrium method to increase inter-class differences between base and novel classes while ensuring balanced performance during fine-tuning for both. Sun et al. [19] utilized contrastive learning and introduced contrastive proposals encoding [19], which further enhances the detector’s ability to distinguish between various classes. These methods calculate loss based on misclassifications in the model’s final output, guiding the model to learn classification knowledge. However, the support set output in meta-learning-based methods contains information about all classes, and enhancing inter-class differences from this can be a promising approach to strengthening inter-class differentiation within the meta-learning framework.

Regarding recent state-of-the-art FSOD methods in RSIs, Cheng et al. [32] introduced Prototype-CNN, which includes a prototype learning network, a prototype-guided RPN, and a detection head tailored for detecting novel objects. Zhang et al. [33] took into account the spatial similarity between the support set and the query set, designing the self-adaptive global similarity module and the two-way foreground stimulator module to enhance the perceptual ability towards novel classes. PAMS-Det [34] utilized the involution operator and shape bias to enhance the classification branch, alongside a multiscale path-aggregation module for refining the regression branch. Moreover, Zhang et al. [35] expanded Generalized FSOD to remote sensing and introduced a comprehensive transfer learning framework.

However, these methods fail to consider a significant issue in FSOD: incompletely annotated objects (IAO) in training images. This poses considerable confusion for the detector and greatly hampers the performance of few-shot object detectors.

### 2.3. IAO Problem

Zhang et al. [36] mentioned the issue of incompletely annotated novel objects, referred to as the IANO problem. To the best of our knowledge, following the N-way-K-shot training principle as outlined in [37] and taking into account all scenarios of label absence during all the training process, we recognize the IAO problem, which includes but not limited to IANO, encompassing:

- IAO-back: During base training, unannotated instances of novel class objects are treated as background.
- IAO-novel: During fine-tuning, unannotated instances of novel class objects may arise.
- IAO-base: During fine-tuning, unannotated instances of base class objects may occur.

Li et al. [38] were the pioneers in identifying the IAO issue in their research, highlighting the potential presence of unlabeled novel objects within the base set images. They devised a network that generates pseudo-labels corresponding to these images, which are then utilized to detect potential novel objects and adjust the loss calculation for the RoI head accordingly. In a similar vein, Qiao et al. [39] emphasized the existence of the IAO challenge, particularly when multiple novel objects are present in a single image. To address this concern, they proposed a label calibration method. This method recalibrates the predicted objects of background objects based on their predicted confidence scores, assigning lower weights to unannotated novel objects during the loss calculation to mitigate their detrimental impact. Based on two-stage object detectors, Zhang et al. [36] utilized advanced self-training techniques not just for the bounding box classification head, but also for the RPN, further addressing the IAO problem. Subsequently, Liu et al. [14] extensively investigated techniques for performing semi-supervised object detection directly on uncurated and unlabeled data. In the meantime, Liu et al. [15] introduced a novel label-consistent classifier that effectively utilizes unannotated new class objects within the base class, addressing the IAO-back and IAO-novel problems.

In general, the aforementioned methods aim to address the IAO-back and IAO-novel issues. Whether from the perspective of label correction or training mode, their primary focus is on reducing the confusion caused by unannotated instances. However, it is crucial to acknowledge that during training, labels and actual objects are not perfectly aligned. We consider the IAO-novel and IAO-base issues, but instead of adding pseudo-labels to alleviate their confusing effects, we employ DCS to remove unlabeled objects from the images. Both approaches aim to mitigate the impact of labeling errors. However, the addition of pseudo-labels leads to inconsistency in the number and categories of labels in each training batch, resulting in sample imbalance and affecting the detector’s decision making for certain classes. Additionally, the increase in the number of labels implies greater computational resource consumption per training iteration, contradicting the lightweight requirements of real sensors. Moreover, it is unreliable for the detector to independently judge whether an object is unannotated, as the model’s understanding may not be sufficient in such cases. Instead of relying on the model’s randomness to determine unannotated objects, it is more practical to provide it with balanced and definite inputs. This approach is cost-effective and feasible, because the matter lies in the fine-tuning stage, during which only a small amount of data is required.

### 2.4. Revisiting YOLOv9 (GELAN-C)

**Figure 4:**
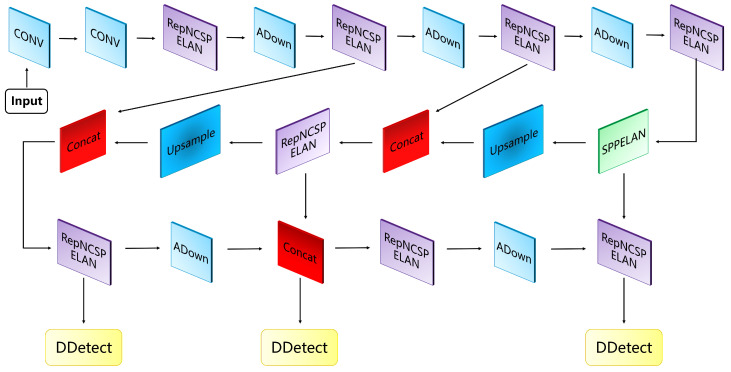
Schematic diagram of overall structure of YOLOv9 (GELAN-C) [20].

YOLOv9 [20], being one of the most cutting-edge detectors, stands out with its ultra-fast inference speed and accurate detection performance. Wang et al. [20] designed GELAN, utilizing only traditional convolutions, which achieves higher parameter efficiency compared to state-of-the-art deep convolutional designs, while demonstrating significant advantages in being lightweight, fast, and accurate. We fully considered the advantages of YOLOv9 [20] and ultimately concluded that the GELAN-C version is the most suitable to be adapted as a few-shot object detector for remote sensing images. The overall structure of YOLOv9 (GELAN-C) is shown in Figure 4.

**Figure 5:**
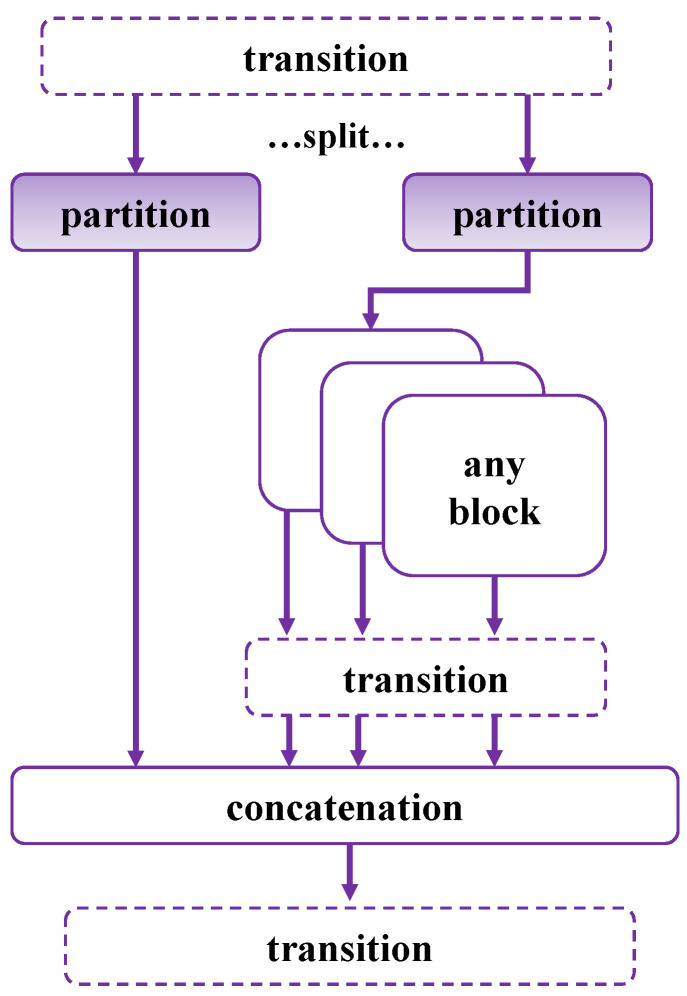
Schematic diagram of GELAN (RepNCSPELAN).

The main features and components of GELAN [20] include the CSP-ELAN block, which integrates the CSPNet (Cross Stage Partial Network) structure into the ELAN (Efficient Layer Aggregation Network) foundation, as shown in Figure 5. This structure can utilize various computational blocks, such as Bottleneck or ResBlock, to enhance computational efficiency. GELAN optimizes its network structure through carefully designed gradient path planning, allowing for more effective propagation and aggregation of feature information from different levels. Its lightweight design focuses on minimizing computational load and parameter count while maintaining detection performance, making it suitable for resource-constrained devices. Despite its emphasis on lightweight design, GELAN [20] achieves high-precision object detection, comparable to more complex network architectures. Therefore, we are committed to making it effectively finish FSOD tasks while maintaining its advantage of fast inference.

## 3. Methods

### 3.1. Problem Setting

FSOD aims to train a detection model on a dataset with base classes. This allows it to detect objects in images with new classes, even with few annotated samples. A meta-learning-based detector is trained to glean meta-knowledge from a vast amount of detection tasks sampled from base classes, enabling it to generalize effectively to novel classes. Each sampled task is termed an episode, where an episode E consists of a collection of support images S and a set of query images Q. During each episode, the support images serve as training samples, instructing the model on how to tackle the given task, while the query images act as test samples, assessing the model’s performance on the task.

For a remote sensing dataset encountering the FSOD problem, we approach it as follows. Following a methodology akin to the fine-tuning approach [36], our meta-learning mode entails two primary stages: base class training and novel class (meta) fine-tuning. Initially, as delineated in [40], we partition the dataset into base class data $D_{base}$ and novel class data $D_{novel}$. Here, $D_{base}$ comprises image data $I_{m}$ and the corresponding label $A_{m}$, denoted as

(1) $$D_{base}=\left\{I_{m}^{i},A_{m}^{i}\right\},m=1,2,3,\ldots ,N_{B},$$

where *m* represents the class sequence, *i* symbolizes the image sequence, and $N_{B}$ denotes the number of base classes. $D_{novel}$ is similarly structured, expressed by

(2) $$D_{novel}=\left\{I_{m^{\prime }}^{j},A_{m^{\prime }}^{j}\right\},m^{\prime }=N_{B}+1,N_{B}+2,\ldots ,N,$$

where $m^{\prime },I_{m^{\prime }},A_{m^{\prime }}$ have the same meanings as represented in $D_{base}$ and *j* symbolizes the image sequence, while *N* denotes the total number of classes. And, there must be

(3) $$\left\{A_{m}^{i}\right\}\cap \left\{A_{m^{\prime }}^{j}\right\}=⌀,$$

meaning that there is no overlap between the labels of the novel classes and the base classes.

During base class training, we form both the query set $Q_{base}$ and the support set $S_{base}$ from $D_{base}$, which can be expressed by

(4) $$\begin{array}{c} Q_{base}\subset \left\{I_{m}^{i},A_{m}^{i}\right\},\\ S_{base}\subset \left\{I_{m}^{i},M_{m}^{i}\right\}, \end{array}$$

where $M_{m}$ denotes the label mask images corresponding to the images. Both the query set and the support set utilize all the base class data. Subsequently, in novel class fine-tuning, adhering to the N-way-K-shot principle, we select K objects and labels for each class for $Q_{novel}$ and $S_{novel}$, which can be expressed as

(5) $$\begin{array}{c} Q_{novel}\subset \left\{\left\{I_{m}^{i},A_{m}^{i}\right\}\cup \left\{I_{m^{\prime }}^{j},A_{m^{\prime }}^{j}\right\}\right\},\\ S_{novel}\subset \left\{\left\{I_{m}^{i},M_{m}^{i}\right\}\cup \left\{I_{m^{\prime }}^{j},M_{m^{\prime }}^{j}\right\}\right\}. \end{array}$$

Assuming $F_{i}$ represents the untrained model, it adheres to the following paradigm throughout the entire training:

(6) $$F_{i}\overset{S_{base}}{\underset{Q_{base}}{\to }}F_{base}\overset{S_{novel}}{\underset{Q_{novel}}{\to }}F_{final},$$

where $F_{base}$ denotes the model after base training and $F_{final}$ inherits the parameters of $F_{base}$, obtained through fine-tuning with $Q_{novel}$ and $S_{novel}$.

### 3.2. Framework Overview

**Figure 6:**
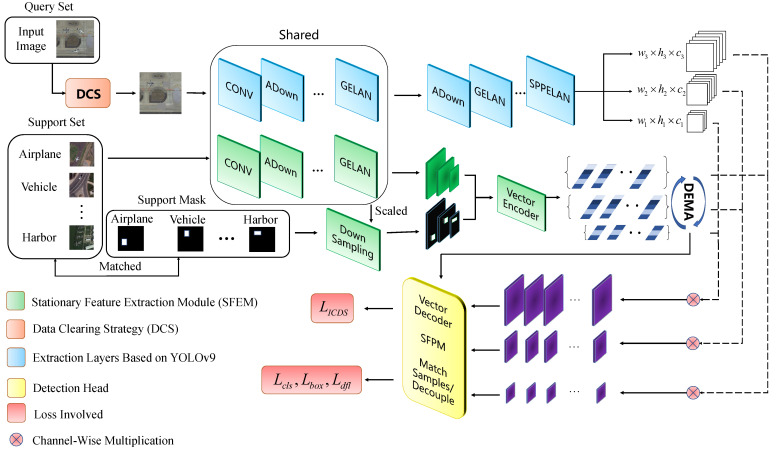
Schematic diagram of the proposed B-FSDet’s overall structure.

The overall structure of B-FSDet is shown in Figure 6. Built upon the YOLOv9 [20] framework, our endeavor focuses on the transformation of efficient single-stage detectors into robust few-shot detectors, while addressing diverse challenges inherent in the practical utilization of RSIs. B-FSDet comprises SFEM, SFPM, DCS, feature extraction layers based on YOLOv9 [20], and a detection head. The training process follows the meta-learning paradigm outlined in [11,12], involving base class training followed by fine-tuning on new classes. During base class training, query set images are processed through the YOLOv9-based feature extraction layer, while support set images undergo shared feature extraction layers before vector encoding. All specific network architecture parameters based on YOLOv9 are available in [20] and Section 2.4, and specific implementation of other modules is detailed in later sections. The encoded vectors then undergo the DEMA operation with previously encoded vectors, followed by channel-wise multiplication with features of the query sets to obtain fused features, which are then inputted into the detection head for loss calculation. In the fine-tuning phase, the query set images undergo DCS to remove unannotated objects before being processed and the operations on the support set remain consistent with the base class training phase. During prediction, SFPM utilizes the stationary support set vectors obtained during training for inference, enabling a further reduction in model parameters.

### 3.3. Few-Shot Data Clearing Strategy (DCS)

**Figure 7:**
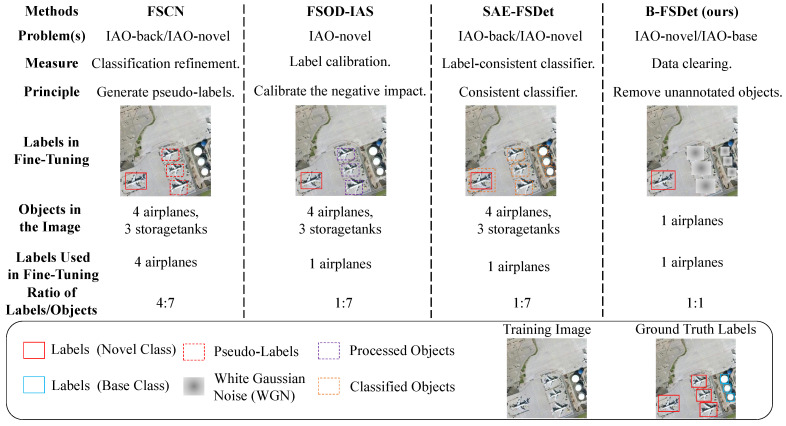
Comparison schematic between our proposed method DCS and others. Here, FSCN is derived from [38], FSOD-IAS from [39], and SAE-FSDet from [15]. Our method contributes to making the number of objects in an image match the number of labels. Note that the illustration is solely for demonstration purposes, and the specific method may not necessarily target RSIs.

As previously discussed, the issues of IAO-novel and IAO-base have a significant impact on the detector’s performance. Previous research has aimed to mitigate confusion for the detector by assigning pseudo-labels to unannotated objects or reduce the impact of the noisy labels [38,39]. In a similar manner, a consistent label classifier is proposed to make the labels more consistent during base training and fine-tuning [15], as shown in Figure 7. However, the inclusion of pseudo-labels is contingent upon the current performance of the network and the distribution of objects within the current batch. Consequently, pseudo-labels are often random and not entirely accurate across all classes. This imbalance in samples can lead to a decrease in detection accuracy for certain classes. Similar methods also rely on the current performance of the model’s classifier to make judgments, so they cannot achieve complete accuracy. The root cause of the IAO problem lies in data imbalance, stemming from both the data collection and data processing. Instead of focusing solely on enhancing the model’s ability to detect unannotated objects, it is more effective to address data imbalance directly through data clearing.

Hence, we propose a simple and effective data clearing strategy (DCS) aimed at FSOD. This method focuses on removing redundant objects from training images rather than adding missing parts to training labels. This process is only conducted during the fine-tuning stage when the data volume is extremely low. It is both feasible and yields significant improvements in performance, aligning with the training principles of FSOD.

Specifically, DCS comprises four steps. Firstly, identify the missing labels for each image during fine-tuning. For example, if an image contains objects $\left\{O_{i}\right\}$ with corresponding labels $\left\{L_{i}\right\}(i=0,1,2,\ldots ,P;P$ denotes the number of objects included), but in the fine-tuning dataset, only the label $L_{k}$ is provided, then the missing labels $\left\{L_{j}\left(j\neq k\right)\right\}$ corresponding to objects $\left\{O_{j}\right\}$ are determined. There should be

(7) $$\begin{array}{c} \left\{L_{j}\right\}\subset \left\{L_{i}\right\},\\ \left\{O_{j}\right\}\subset \left\{O_{i}\right\}. \end{array}$$

Secondly, locate the objects corresponding to the missing labels. In digital images, each pixel is inherently discrete. $O_{j}\left(x,y\right)$ represents a two-dimensional function, denoting the object portion of the entire image. Thirdly, replace these objects with white Gaussian noise (WGN). Finally, restore the annotated object to its background. It is important to consider that targets in RSIs may overlap, and losing annotated objects along with some background information is not desirable. Therefore, annotated instances and their surrounding 10 pixels are preserved and restored.

The universality of WGN in randomness enables its substitution for unannotated objects, making it adaptable to any target. Coupled with the loss function ignoring negative samples, it effectively mitigates the noise impact while addressing the issues of IAO-novel and IAO-base. Let $WGN∼N(\mu ,\sigma^{2})$, the pixel values of each point of the target with missing labels are replaced through random sampling from WGN. WGN is a random variable with a probability density function given by

(8) $$f\left(x\right)=\frac{1}{\sqrt{2\pi \sigma }}e^{-\frac{x-\mu }{2\sigma^{2}}}.$$

In DCS, we let μ=0.5,σ=5.

### 3.4. Stationary Feature Extraction Module (SFEM)

A notable drawback of the meta-learning-based method is the difficulty in designing detectors. FSODM [11] has demonstrated certain rationality in supporting set output features to assist query set feature learning in vector form. This approach is efficient and parameter-free, yet the variance in the output vectors is relatively large, making it hard to represent the whole class. Based on [11,20], we redesign the feature extraction module and propose a loss-based dynamic exponential moving average (DEMA) method, where the output vectors are influenced not only by the current batch input support set images but also by the output vectors from previous batches. Therefore, during the later stages of training, the vectors originating from all objects within the support set can adequately represent the majority of class features. In this process, the task is accomplished solely with common convolutional layers, in accordance with the lightweight design of the model. The extraction part is detailed in Table 1.

**Figure 8:**
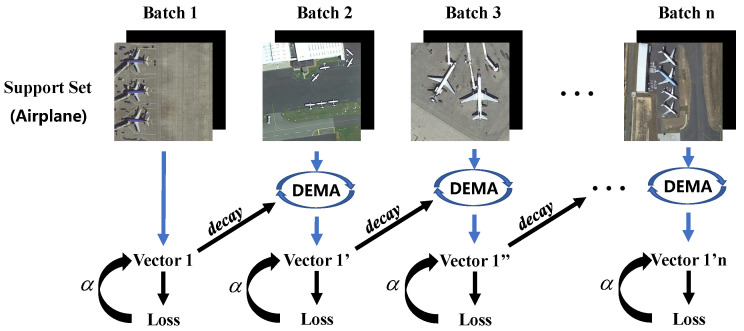
Schematic diagram of DEMA operation process. The final output vector is associated with all support set objects, resulting in more stable auxiliary effects on the query set. Only one scale of processing is illustrated for ease of visualization.

As shown in Figure 8, the specific procedure of DEMA involves recording the encoding vectors $V_{1},V_{2},V_{3}$ during the last batch training. Subsequently, in the next batch, the output vectors are updated by

(9) $$\begin{array}{c} V_{1}^{\prime }=decay\times V_{1}+\left(1-decay\right)\times V_{1}^{\prime },\\ V_{2}^{\prime }=decay\times V_{2}+\left(1-decay\right)\times V_{2}^{\prime },\\ V_{3}^{\prime }=decay\times V_{3}+\left(1-decay\right)\times V_{3}^{\prime }, \end{array}$$

where $V_{1}^{\prime },V_{2}^{\prime },V_{3}^{\prime }$, respectively, represent the current vector’s output, and decay is the dynamic factor scaled by the loss L in the last batch, as expressed by

(10) decay(L)=L×α.

In SFEM, α=0.01. During the experiments, the typical range of the total loss L is between 10 and 40, resulting in the update weight decay accounting for approximately 10% to 40%. Further details of L are introduced in the next part. The behavior of the decay weight is dynamically adjusted based on the loss of the current iteration. If the loss for the current iteration is significant, indicating that the quality of the current batch is not satisfactory, the decay weight will increase. This results in placing more emphasis on the weight of previous DEMA results to stabilize the learning process. Conversely, if the loss is lower, implying a higher quality batch, the decay weight will decrease, allowing the model to adapt more quickly to new information.

As training progresses, particularly in the later stages, $V^{\prime }$ comes to represent relatively stable class centroids. This stability is crucial for ensuring that the model’s predictions become more consistent and reliable over time, reflecting the accumulated knowledge from previous iterations. By dynamically adjusting the decay weight, the model effectively balances between integrating new data and retaining the robustness of learned features, ultimately leading to improved performance and generalization.

### 3.5. Loss Computation

During training, SFEM reduces the variance in the output vectors, making the feature vectors provided by the support set more stable and reliable. However, in RSIs, inter-class differences are often minimal. To increase the differentiation of each class component in the support set output vectors while maintaining stability, we propose the inter-class discrimination support loss (ICDSL). In addition to channel fusion with the query set, the vectors encoded by the support set directly enter the detection head. We integrate a vector decoder into the detection head and apply the cross-entropy loss (CEL) to the decoded results to enhance the detector’s ability to distinguish between classes.

The whole loss is calculated by

(11) $$L=g_{b}L_{box}+g_{c}L_{cls}+g_{f}L_{dfl}+\lambda L_{ICDS},$$

where $L_{box},L_{cls},L_{dfl}$ are based on [20], and $L_{ICDS}$ is based on the CEL. $g_{b},\;g_{c},\;g_{f}$ are fixed hyperparameters with $g_{b}=1,\;g_{c}=0.5,\;g_{f}=1.5$, while λ represents the gain of $L_{ICDS}$, which is discussed in detail in subsequent experiments. Suppose the aggregation features $AF_{qs}$ before the detection head are expressed by

(12) $$\begin{array}{c} AF_{qs}=R^{w_{qs}\times h_{qs}\times c_{qs}}, \end{array}$$

where $w_{qs},h_{qs},c_{qs}$, respectively, represent the width, height, and channels of the images (for the sake of simplicity, only one scale is presented), and qs represents the sources of fused features, where *q* denotes those from the query set and *s* denotes those from the support set.

**Figure 9:**
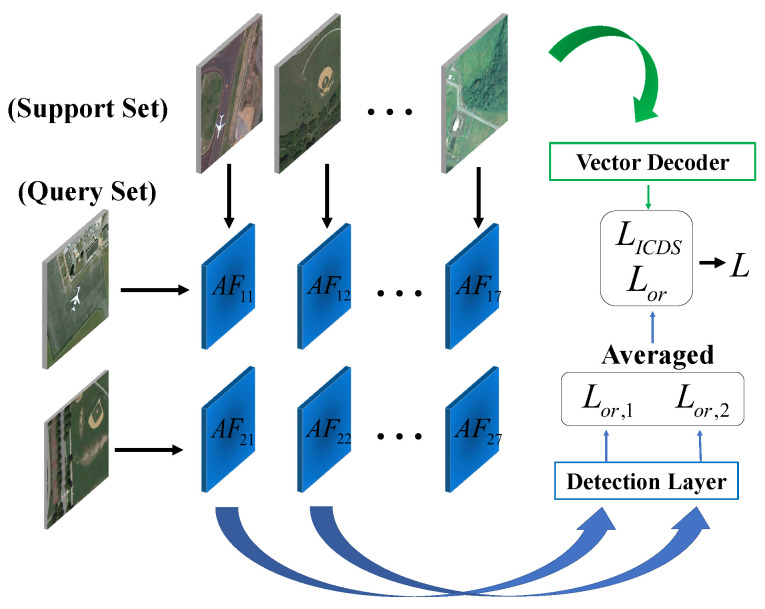
The whole loss computation diagram. The object of calculation for $L_{or}$ is the fusion results of the query set and the support set that match with each other. The object of calculation for $L_{ICDS}$ is the encoding result of the support set. Suppose batch = 2 in the figure.

As shown in Figure 9, when calculating $L_{or}$, the detection layer first filters out the fused features that match between the support set and the query set, and then, decouples them, calculating the corresponding $L_{or,1}$ and $L_{or,2}$. It is evident that non-matching fused features $AF_{12}$ and $AF_{21}$ are introduced during the computation of $L_{or}$. We smooth the calculated loss by averaging the loss involved; hence, the final $L_{or}$ is expressed by

(13) $$L_{or}=\frac{1}{batch}\left(L_{or,1}+L_{or,2}+\ldots +L_{or,batch}\right).$$

We consider the non-matching fused features as noise handled by data augmentation, enhancing the model’s robustness against interference.

**Figure 10:**
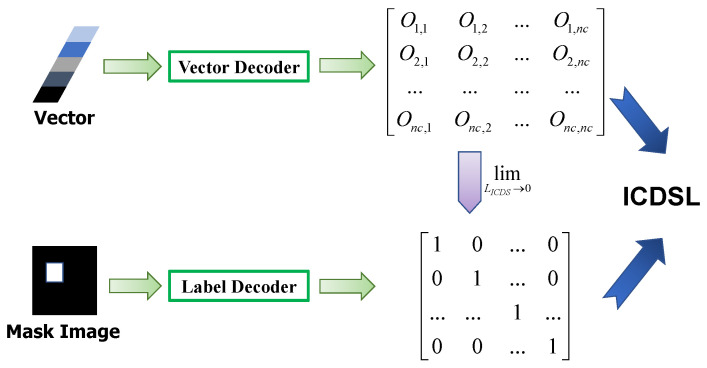
Diagram of the principle of $L_{ICDS}$ calculation. Only one scale of processing is illustrated for ease of visualization.

As for $L_{ICDS}$, firstly, let the output vectors be $V_{1},V_{2},V_{3}$, representing the three scales of features. *D* denotes the decoder operator and we have the decoding output

(14) $$\begin{array}{c} X_{1}=D\left(V_{1}\right)=\left\{O_{i,j}\right\},\\ X_{2}=D\left(V_{2}\right)=\left\{O_{i,j}^{\prime }\right\},\\ X_{3}=D\left(V_{3}\right)=\left\{O_{i,j}^{\prime \prime }\right\}, \end{array}$$

where $O_{i,j},O_{i,j}^{\prime },O_{i,j}^{\prime \prime }\left(i=1,2,3,j=1,2,3,\ldots ,nc\right)$ represents the class score after the linear transformation *D* of the three scales. Then, we introduce CEL to enhance inter-class disparities within RSIs. This enables the model to effectively discern subtle differences between different classes, thereby improving the classification performance. As illustrated in Figure 10, by penalizing incorrect classifications and rewarding correct ones based on the logarithmic difference between predicted and ground truth class probabilities, the model is incentivized to learn more discriminative features representative of each class. $L_{ICDS}$ is calculated by

(15) $$X_{j}=softmax\left(X_{j}\right),$$

(16) $$L_{ICDS}=-\frac{1}{N}\sum_{j=1}^{3}\sum_{i=1}^{N}GT_{i}\times log\left(X_{j}\right),$$

where GT represents the ground truth classes.

### 3.6. Stationary and Fast Prediction Method (SFPM)

**Figure 11:**
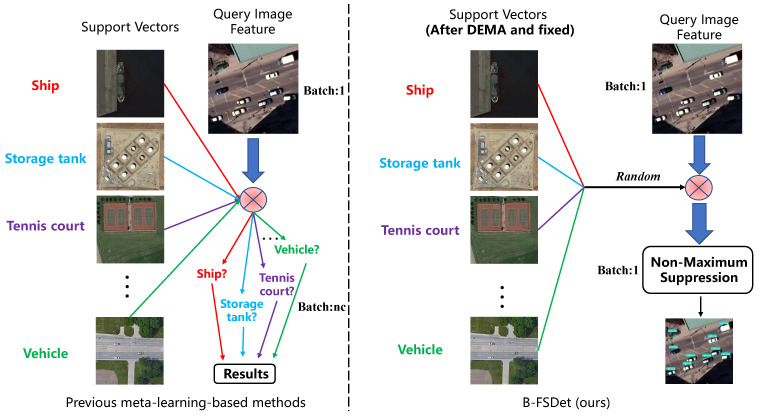
Schematic diagram comparing SFPM with the prediction methods of previous meta-learning-based few-shot object detectors [11,12].

In both the CAA [16] and CSA [12] feature aggregation methods, previous meta-learning-based few-shot detectors entail the fusion of image output features with all classes in the support set during prediction. Subsequently, the result corresponding to the matched support set class is obtained from the fused features, as shown in Figure 11.

Regardless of whether the auxiliary features from the support set are constant or generated in real time during prediction, this approach still slows down the model’s prediction speed. Additionally, due to significant inter-class differences, the vectors may not necessarily match the objects in query set images perfectly. We propose a fast and accurate prediction method SFPM, as shown in Figure 11. During prediction, it is unnecessary to fuse every vector from the support set with the query set; instead, only one is randomly selected. This implies that B-FSDet can effectively distinguish between different classes, thanks to the support of ICDSL and the introduction of non-matching scenarios during feature fusion at training time. In subsequent experiments, we demonstrate that SFPM achieves significantly faster inference speeds compared to previous methods, without compromising much detection accuracy.

## 4. Experiments and Results

To comprehensively demonstrate the efficacy of our proposed detector and methodology, we conducted experiments on the NWPU.v2 [41] and DIOR [42] datasets. The experimental outcomes substantiate the effectiveness of our approach.

### 4.1. Dataset

#### 4.1.1. NWPU VHR-10.v2 Dataset

Containing a total of 10,000 VHR satellite images, the NWPU.v2 dataset [41] encompasses various urban and rural scenes, capturing a wide array of environmental contexts and object types. Each image is meticulously labeled, with bounding boxes delineating the precise locations of objects belonging to ten distinct categories, including airplanes, ships, storage tanks, baseball diamonds, tennis courts, basketball courts, ground track fields, harbors, bridges, and vehicles. The images in the NWPU.v2 dataset exhibit resolutions ranging from 0.1 to 2 m per pixel, ensuring a high level of detail, suitable for object detection tasks. This variation in resolution reflects the diversity of sensors and imaging platforms employed in remote sensing applications.

#### 4.1.2. DIOR Dataset

The DIOR dataset [42] represents a significant breakthrough in Earth observation and sensors. It addresses limitations found in previous datasets, distinguished by its extensive scale, diversity, and complexity. Comprised of more than 20,000 high-resolution images and a total of more than 192,000 object instances spanning 20 categories, DIOR maintains consistency with images all sized at 800 × 800 pixels. Characterized by its intricate background and imbalanced sample distribution, the DIOR dataset garners considerable attention in the field of remote sensing, particularly in the realm of FSOD.

### 4.2. Experimental Setting

Following the experimental setup outlined in [11] and the conventions adopted by numerous SOTA few-shot detectors, we partitioned the categories of the NWPU.v2 and DIOR datasets. The categorization results are delineated in Table 2 and Table 3.

The detailed experimental configuration is outlined as follows: The initial learning rate is configured to 0.01, with SGD optimizer and Adam optimizer set to 0.01 and 0.001, respectively. The final learning rate remains constant at 0.01 throughout training. Momentum is stipulated as 0.937, representing either the momentum parameter for SGD or the $\beta_{1}$ parameter for Adam. Weight decay is established at 0.0005. Warmup epochs, totaling 3.0 epochs, are designated for initial training, with the provision for fractional specification. Initial momentum during warmup is fixed at 0.8, while the initial bias learning rate during warmup is defined as 0.1. Training iterations are executed with a batch size of 6, utilizing an NVIDIA GeForce GTX 2080Ti GPU. During inference, the confidence threshold and intersection-over-union threshold in non-maximum suppression are set to 0.01 and 0.7, respectively.

Fine-tuning datasets of three independent experiments, encompassing both base and novel classes, are randomly generated. The results presented are averaged from these experiments. This consistent protocol is applied to all other methodologies in our experimental evaluations. Wolf et al. [43] proposed that enlarging the sampling scope of base class data to the entire training dataset in the fine-tuning process would contribute to accuracy enhancement. To ensure fairness in comparison, we continue to apply the sampling principle of TFA [37] when obtaining the fine-tuning dataset.

### 4.3. Comparing Methods and Evaluation Metrics

In order to demonstrate the effectiveness of our proposed B-FSDet, we conduct a comparative evaluation of its performance against various SOTA methods. The assessed FSOD methods include FsDetView [10], P-CNN [32], TFA [37], SAGS&TFS [33], G-FSDet [35], and SAE-FSDet [15]. The accuracy data for TFA [37], FsDetView [10], and G-FSDet [35] are obtained from [35]; the accuracy data for P-CNN [32] on the NWPU.v2 dataset is from [35], and for the DIOR dataset is from [32]. The accuracy data for both SAGS&TFS [33] and SAE-FSDet [15] methods are obtained directly from the source articles. For the inference speed data, except for SAE-FSDet provided by [15], all other data are obtained using the same device (an NVIDIA GeForce GTX 2080Ti GPU).

In the assessment of few-shot object detectors, some studies [35] consider both the accuracy of novel classes and base classes, while some [15,33] solely focus on novel class accuracy. We adopt the former way of evaluation, considering the detector’s performance through both the base and novel classes, and dividing classes into base and novel categories based on Table 2 and Table 3. AP50 (referred to as AP hereafter) serves as the prevalent accuracy evaluation metric in the field of object detection. In FSOD, base class performance is typically measured using bAP, while nAP is used to assess the performance of novel classes. Suppose class $i(i=1,2,\ldots ,N_{B})$ belongs to base classes, and class $j(j=N_{B}+1,N_{B}+2,\ldots ,N)$ belongs to novel classes (*N* denotes the number of the training classes), bAP and nAP can be expressed by

(17) $$\begin{array}{c} bAP=\frac{1}{N_{B}}\sum_{i=1}^{N_{B}}AP_{i}, \end{array}$$

(18) $$\begin{array}{c} nAP=\frac{1}{N-N_{B}}\sum_{j=N_{B}+1}^{N}AP_{j}. \end{array}$$

In the subsequent analysis, we also utilize mAP, expressed by

(19) $$\begin{array}{c} mAP=\frac{1}{N}\sum_{k=1}^{N}AP_{k}. \end{array}$$

### 4.4. Results on NWPU VHR-10.v2 Dataset

The performance of our proposed B-FSDet on the NWPU.v2 dataset compared with SOTA detectors is shown in Table 4. In the table, items highlighted in red and bold represent the best performance, while those highlighted in blue and bold indicate the second-best performance. Subsequent tables are annotated following this principle.

From Table 4, it is apparent that TFA [37], as a classic detector employing a fine-tuning strategy, effectively preserves base class knowledge. It demonstrates commendable performance in base class accuracy across various scenarios, consistently achieving a bAP exceeding 89%. SAE-FSDet [15], which addresses the IAO-novel problem, primarily focuses on the detector’s capacity to learn new classes in the NWPU.v2 dataset. It exhibits substantial advantages in nAP across all scenarios, surpassing previous detectors by a significant margin. G-FSDet [35], addressing both the forgetting of base class knowledge and the acquisition of new class knowledge, demonstrates notable advantages in overall performance. Our proposed B-FSDet, designed to address both the IAO-novel and IAO-base problems, outperforms the mentioned few-shot object detectors in most scenarios. Notably, in the split 1 setting, B-FSDet achieves an nAP of 75.27%, significantly surpassing existing SOTA detectors. Moreover, in the 10-shot and 20-shot scenarios, both bAP and nAP exceed 90%. Whether considering current SOTA methods or B-FSDet, the nAP performance in split 1 consistently exceeds that in split 2. This disparity arises from the varying scales and learning difficulties of novel class objects under different split settings. In split 1, novel classes like “airplane” exhibit smaller inter-class differences, making the learning process easier. Conversely, split 2 includes categories like “ground track field” with larger scales and more complex knowledge, increasing the difficulty of learning under few-shot conditions. Overall, B-FSDet demonstrates SOTA performance on the NWPU.v2 dataset.

**Figure 12:**
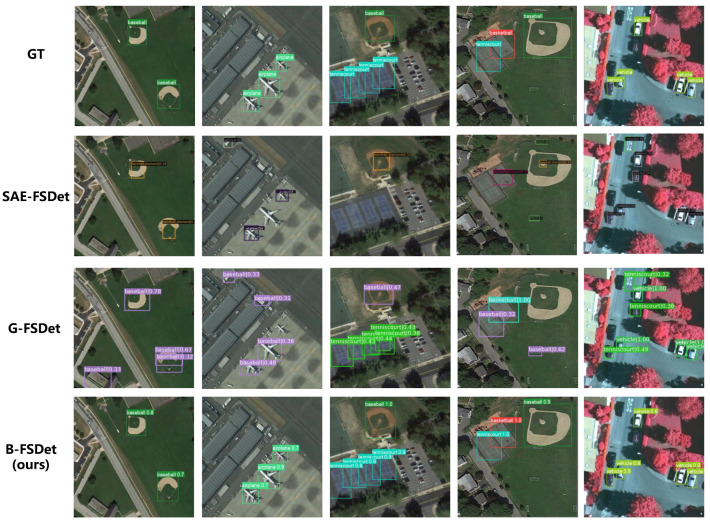
Visualization results compared with some SOTA methods on the NWPU.v2 dataset under the 10-shot setting. Note that here, for the sake of word abbreviation, “baseball” actually refers to “baseball diamond”. The novel classes include airplane, tennis court, and baseball diamond.

The visualization results of B-FSDet compared with SAE-FSDet [15] and G-FSDet [35] under the 10-shot setting on the NWPU.v2 dataset can be seen in Figure 12. The label-consistent classifier of SAE-FSDet [15] significantly reduces the confusion between novel classes and base classes; however, it suffers from a high rate of missed detections. G-FSDet [35] on the other hand, does not consider the IAO issue, making it prone to false detections. In contrast, B-FSDet exhibits superior performance, demonstrating strong perception and recognition capabilities for both novel and base classes.

Table 5 presents the accuracy of B-FSDet for each category on the NWPU.v2 dataset. It can be observed that B-FSDet achieves high detection accuracy for most categories, with only a few categories, such as bridges and vehicles, showing slightly lower accuracy. This variation is attributed to the diverse scales of different objects, which necessitate varying amounts of sample data to effectively extract class features. Consequently, the accuracy for some categories may be lower.

### 4.5. Results on DIOR Dataset

A performance comparison between B-FSDet and other advanced few-shot object detectors on the DIOR dataset is presented in Table 6. Similar to its performance on the NWPU.v2 dataset, TFA [37] demonstrates significant superiority in terms of bAP, exhibiting commendable performance across various splits and shot settings. SAGS&TFS [33] and SAE-FSDet [15] focus on improving nAP performance, resulting in some enhancements in nAP compared to previous methods across various scenarios. While SAE-FSDet takes into account the IAO-novel issue, the efficacy of its proposed consistency classifier is contingent upon the training conditions of the detector itself. As a result, its ability to alleviate the IAO problem is somewhat random. Consequently, its accuracy does not rank as the highest among numerous SOTA methods. G-FSDet [35] continues to emphasize both bAP and nAP performance, showcasing good overall performance across different scenarios. Similarly, our proposed B-FSDet considers both bAP and nAP performance, achieving SOTA performance in most settings. In the split 1 setting, B-FSDet outperforms other methods in all scenarios, with both nAP and bAP surpassing the second-ranked method by 4% to 8%. In the split 3 setting, B-FSDet demonstrates significant superiority in bAP, surpassing the second-ranked method by approximately 11%, while exhibiting comparable performance to TFA [37] in terms of base class performance. In the remaining split settings, except for 3-shot in the split 4, B-FSDet demonstrates similarly commendable performance. In the context of the split 4 3-shot scenario, it is evident that the nAP of B-FSDet is marginally lower than that of SAE-FSDet [15] and G-FSDet [35]. This discrepancy arises from the fact that, under the split 4 setting, the novel class objects, including express service area and stadium, tend to have larger scales and encompass more intricate information. Based on Faster R-CNN [4], SAE-FSDet [15] and G-FSDet [35] manage to mitigate this issue to some extent owing to the reinforcement provided by pre-selected proposal boxes. However, B-FSDet maintains a substantial advantage in preserving knowledge of base classes, with its bAP exceeding that of other methods by over 5%.

It is evident that current SOTA detectors display considerable performance discrepancies across different split settings, whereas B-FSDet exhibits relatively minor variations in performance across different scenarios. This is partly attributed to SFEM and DCS effectively balancing the inherent differences in the data and enhancing the detector’s robustness.

**Figure 13:**
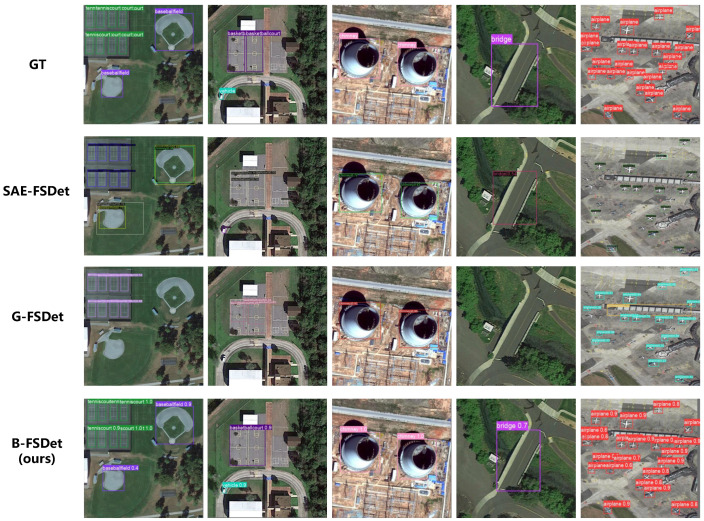
Visualization results compared with SOTA methods on the DIOR dataset under the ten-shot setting. The novel classes include baseball field, basketball court, bridge, and chimney.

The visualization results of B-FSDet compared with SAE-FSDet [15] and G-FSDet [35] on the DIOR dataset under the 10-shot setting are shown in Figure 13. SAE-FSDet [15] addresses the IAO-novel issue; however, its label-consistent classifier cannot guarantee the correctness of all classification results. Consequently, the IAO problem is not entirely resolved, leading to instances of missed detections and duplicate detections of novel classes. G-FSDet [35], on the other hand, exhibits excellent overall performance but does not account for the IAO issue. As a result, it experiences slightly more missed detections of novel classes compared to SAE-FSDet [15], although it demonstrates better retention of base class knowledge. In contrast, B-FSDet focuses on both the IAO issue and the preservation of base class knowledge, providing a significant performance advantage. However, B-FSDet still faces challenges when detecting closely adjacent objects. For instance, as shown in the second graph in Figure 13, B-FSDet detects two basketball courts, but their positions nearly overlap, causing the detector to identify them as a single object and even resulting in duplicate detection.

Table 7 presents the accuracy of B-FSDet for each category in the DIOR dataset. It can be seen that B-FSDet maintains consistent accuracy for base classes across different shot settings, indicating its effectiveness in retaining base class knowledge when handling large datasets. As the amount of samples increases, the accuracy for most novel classes improves significantly, demonstrating B-FSDet’s strong learning capability for new classes. For some categories, the accuracy does not improve with an increased sample amount. We attribute this to the additional samples not providing effective feature information and having substantial overlap with previously existing samples. Additionally, the DIOR dataset features targets with more diverse scales and varying levels of complexity, leading to greater differences in accuracy across categories compared to the NWPU.v2 dataset.

### 4.6. Ablation Study

In order to comprehensively demonstrate the effectiveness of the modules or methods we propose, we conduct ablation experiments on the split 1 setting of the NWPU.v2 dataset. The modules or methods we introduce include SFEM, SFPM, DCS, and ICDSL. Initially, SFEM and SFPM are employed to adapt the baseline to a stationary meta-learning-based (S-Meta) few-shot object detector. Subsequently, DCS is integrated into the detector to mitigate the confusion caused by the IAO problem. Finally, ICDSL is applied to further augment the detector’s inter-class discrimination capability. The experimental results are summarized in Table 8.

As we can see, when faced with small-scale datasets such as NWPU.v2, the leading conventional detector YOLOv9 [20] demonstrates good performance. We transformed and improved it to enhance its capabilities in FSOD using S-Meta. The results reveal that all our proposed methods or modules notably enhance the effectiveness in FSOD tasks. In both the 3-shot and 5-shot setting, there is a moderate increase in bAP, and in the meantime, nAP sees an increase of approximately 20%. Likewise, in the 10-shot and 20-shot scenarios, there is a significant improvement across all metrics.

**Figure 14:**
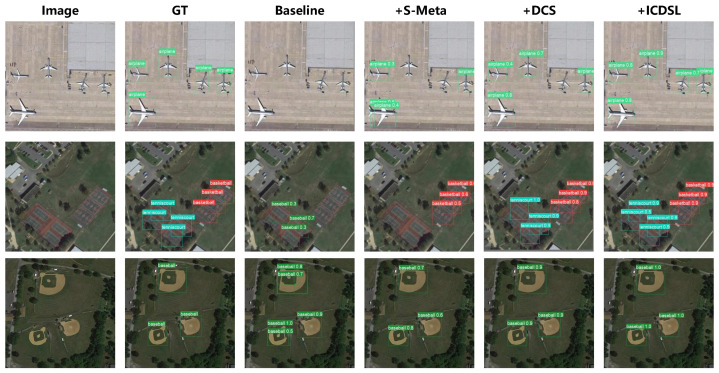
Visualization of ablation experiment results on the NWPU.v2 dataset. The experimental setup in the figure is configured as 10-shot. Note that here, for the sake of word abbreviation, “baseball” actually refers to “baseball diamond”. The novel classes include “airplane”, “tennis court”, and “baseball diamond”.

The visualized results of the ablation experiments on the NWPU.v2 dataset are presented in Figure 14. It is clear that the baseline (YOLOv9 [20]) struggles with accurately detecting new classes, resulting in issues like missed and redundant detections. As we can see, in Figure 14, the baseline exhibits significant instances of missed detections for both the “airplane” and “tennis court” categories, while for the “baseball diamond” category, it produces redundant detections for the two instances. While the S-Meta brings some improvement in addressing redundant detections, the problem of missed detections persists due to the presence of IAO. Integration of DCS helps mitigate the confusion caused by the IAO problem, further enhancing the resolution of missed detections. However, due to significant inter-class differences in RSIs, minor instances of missed detections still occur. With the adjustments of ICDSL, the problem is further addressed.

### 4.7. Study of ICDLS Gain

**Figure 15:**
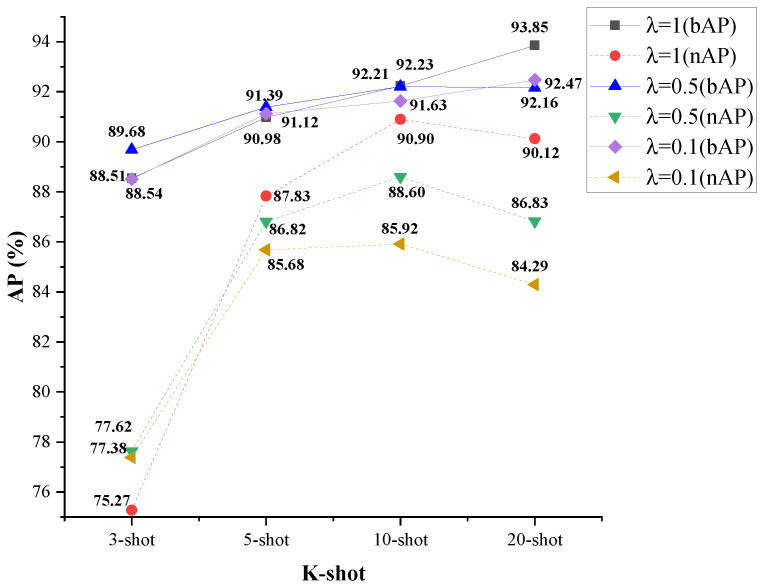
Ablation experiment results on the NWPU.v2 dataset regarding the gain λ of ICDSL.

The specific gain of ICDSL significantly affects the classification capability of the detector. We also investigate the influence of the gain λ on the detector’s performance. Since the typical values (not scaled) for ICDSL are in the range of 10 to 20, while the typical values for the remaining losses (not scaled) are in the range of 10 to 40, we choose gain values of 1, 0.5, and 0.1 to conduct the experiment. The experimental results are shown in Figure 15, which indicate that the detector achieves the best overall performance when λ is set to 1.

### 4.8. Complexity and Inference Time

**Figure 16:**
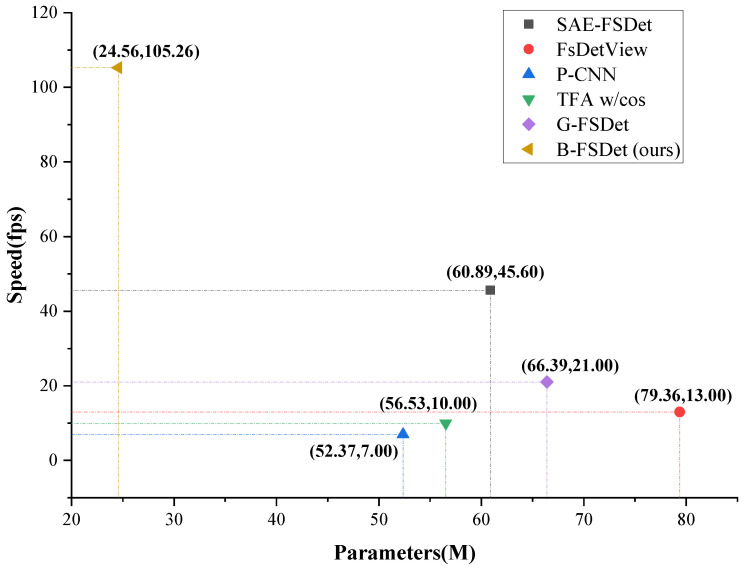
Diagram of the comparison of model parameters and inference speed with SOTA few-shot object detection models. The data of speed and model parameters for the SAE-FSDet [15] method were measured on one NVIDIA GeForce RTX 4090 GPU by [15], while all other data were obtained using one NVIDIA GeForce GTX 2080Ti GPU.

Considering the requirements of real-time systems, we prioritized a lightweight design when developing the detector. A comparison of model parameters and inference speed with numerous state-of-the-art (SOTA) models can be seen in Figure 16. Many few-shot detection models are based on the two-stage detector Faster R-CNN [4], while B-FSDet is based on YOLOv9 [20]. The calculation of frames per second (fps) is based on inference time in the testing phase (the GPU has completed its warmup phase), which includes preprocessing, forward pass, and non-maximum suppression. Originating from the partitioning results of the NWPU.v2 dataset [41], the size of the test images is 400 × 400 pixels. While preserving the advantage of fast inference of one-stage detectors, B-FSODet successfully accomplishes the FSOD task.

As described in Section 3, SFPM accelerates the inference speed of the detector. We also conducted ablation experiments to illustrate its effectiveness, with the results shown in Table 9. As for the baseline YOLOv9 (GELAN-C) [20], its inference time is as low as 9.1 ms, achieving a frame rate of 109.89 fps. The addition of meta-learning mode, while offering some accuracy enhancement, leads to a notable decrease in inference speed. This is attributed to the fact that during the detection layer the batch size increases by a factor of nc (number of classes), resulting in a reduction in inference speed of approximately nc × 0.5 times. In B-FSDet with SFPM, the inference collectively takes around 9.5 ms, achieving an fps of 105.26 on the condition that the accuracy remains high.

To further substantiate the real-time inference capabilities of B-FSDet across different devices, we conduct additional inference speed testing experiments on three other devices. The results are shown in Table 10. It is evident that B-FSDet demonstrates excellent inference speed across four different NVIDIA GPUs, with GPU memory usage around 8 MB, further substantiating the lightweight design of B-FSDet.

### 4.9. Discussion

#### 4.9.1. Few-Shot Object Detectors Still Encounter Challenges When Faced with Large-Scale Datasets

The experimental results clearly demonstrate that our detector achieves SOTA performance on the NWPU.v2 dataset. Even under low-shot conditions, the detector maintains extremely high detection accuracy, with the bAP exceeding 70% in the 3-shot split 1 scenario. However, on the DIOR dataset, although most metrics also surpass those of the current SOTA detectors, there is still room for improvement in detection performance. This can be attributed to several factors. Firstly, the DIOR dataset contains a significantly larger number of training and testing images compared to the NWPU.v2 dataset. Moreover, the DIOR dataset exhibits greater intra-class variation and smaller inter-class variation. And, the scale variation in objects in the DIOR dataset is also much larger. Additionally, although the two-stage training paradigm ensures that during the fine-tuning stage the balance of input sample labels can be maintained, during the base class training stage the number of labels for each class cannot be relatively balanced due to inherent characteristics of the dataset.

#### 4.9.2. The IAO Problem Exerts Negative Guidance during Fine-Tuning in FSOD

In cases where the detector undergoes excessive training epochs or achieves very low loss, a decline in accuracy may occur. In conventional detectors, this is often attributed to model overfitting and dynamic learning rate strategy. In FSOD, this accuracy decline is further exacerbated by the influence of the IAO problem, leading to a more severe decline. Hence, we conducted a detailed experiment to analyze the accuracy during the training process on the NWPU.v2 dataset split 1 setting (10-shot scenario).

**Figure 17:**
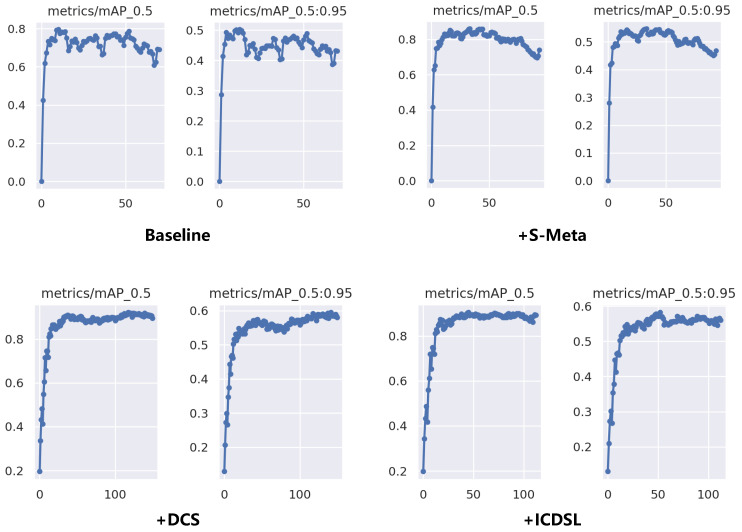
Diagram of the changes in training accuracy in the detailed experiment under the 10-shot setting on the NWPU.v2 dataset.

Figure 17 illustrates the training accuracy for the baseline, the one with S-Meta mode, further enhanced with the DCS, and the one supported by ICDSL. We set epoch = 150 and patience = 60, which means that training automatically stops when the accuracy does not improve compared to the previous 60 epochs. If the accuracy keeps improving, training continues for a maximum of 150 epochs. Under this configuration, the baseline achieves its highest accuracy around the 20th epoch. With the epoch increasing, the accuracy does not consistently increase but surprisingly decreases. The principal cause of this phenomenon is the IAO issue, compounded by the influence of overfitting. During training, the detector receives supervision for labeled objects, enabling it to learn useful knowledge about the targets. However, due to the negative influence of the unlabeled objects, the detector may also learn to classify them as background. Consequently, the reduction in loss guides the detector to focus solely on detecting the labeled objects during training, leading to the abandonment of knowledge about unlabeled objects. Therefore, for detectors not considering the IAO problem, it is advisable to conduct testing every 50 epochs during training to determine whether the detector has entered a state influenced by the negative guidance of the IAO problem.

In the S-Meta paradigm, there is a certain improvement in detector accuracy, accompanied by an increase in convergence rounds. Incorporating S-Meta also reduces the fluctuation in accuracy, further demonstrating its stability enhancement. However, due to the influence of the IAO problem and overfitting, the detection accuracy still exhibits a trend of initially increasing, and then, decreasing. With the addition of the DCS, the IAO problem is alleviated and the accuracy shows a relatively stable increasing trend. Ultimately, ICDSL amplifies inter-class distinctions. However, since its input solely relies on support set vectors untouched by the IAO issue, the enhancement in mitigating the IAO problem is not substantial but there is still an enhancement in accuracy.

#### 4.9.3. Why We Choose WGN in DCS

**Figure 18:**
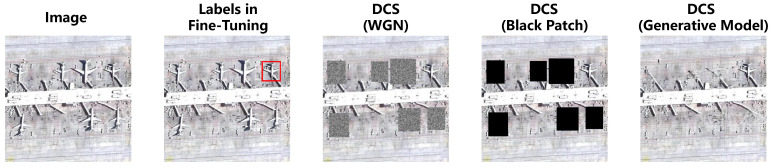
Illustrative diagram of visual results for specific DCS schemes.

In the previous section, we discuss using WGN to replace unannotated objects to alleviate the IAO problem. To justify the employment of WGN, we conduct comparative experiments using other two different methods: pure black patch (BP) replacement and supplementary black patch generation via generative models (GMs) [44]. The visualization of the processed input by WGN, black patch replacement, and generative models is shown in Figure 18. Quantitative detection accuracy results are presented in Table 11.

It is evident that GM and WGN offer more advantages in accuracy compared to the BP method. This is because replacing unannotated objects with pure black blocks does not benefit the detector’s robustness and generalization performance. In contrast, the random nature of WGN significantly enhances the detector’s robustness and GM generates visually reasonable backgrounds using strong prior knowledge, further improving the model’s robustness. The results of GM and WGN are comparable, but employing GM means greater computational complexity. Therefore, WGN is chosen as the final implementation for DCS.

## 5. Conclusions

This paper presents B-FSDet, a few-shot object detector based on YOLOv9 and meta-learning, designed to tackle various challenges encountered in RSIs. Firstly, we introduce the DCS, which effectively filters out incompletely annotated objects from images, ensuring a balanced distribution of true labels and objects and thereby reducing confusion for the detector. Secondly, we propose SFEM and SPFM, constructing a stationary meta-learning mode achieving high detection accuracy while maintaining extremely fast inference speeds. Finally, ICDSL is introduced to increase inter-class differences among target classes, enhancing the detector’s ability to distinguish between classes effectively. The results indicate that B-FSDet achieves a detection accuracy approximately 8% higher than current methods in most scenarios. Specifically, in the NWPU.v2 dataset, under the 3-shot setting, B-FSDet achieves an nAP exceeding 75%, while under the 20-shot setting, both bAP and nAP are close to or exceed 90%. On the DIOR dataset, B-FSDet achieves a bAP exceeding 70% under all settings, while under all split settings with the 20-shot setup, nAP exceeds 40%. Additionally, the experimental data in Figure 16 demonstrate that our proposed B-FSDet achieves an inference speed approximately 2–3 times faster than current SOTA methods.

While B-FSDet has achieved considerable progress, addressing the substantial intra-class variations present in RSIs poses ongoing challenges. Particularly, acquiring comprehensive knowledge of new classes with extremely limited samples remains a significant hurdle. Future efforts will focus on “expanding balanced samples” to enable the detector to acquire more nuanced knowledge of new classes and explore a balanced classifier to match with it. Additionally, a learnable self-attention layer may replace the DEMA strategy to realize better performance.

## Figures and Tables

**Table 1:** The whole framework of SFEM. Notice that the size of the input image is 640×640×3.

Index	Layer	Output Size	Route
0	Conv	320×320×64	Next
1	Conv	160×160×128	Next
2	RepNCSPELAN	160×160×256	7
3	ADown	80×80×256	Next
4	RepNCSPELAN	80×80×512	7
5	ADown	40×40×512	Next
6	RepNCSPELAN	40×40×512	7
7	Mask Integration	$w_{i}\times h_{i}\times {c_{i}\;}^{1}$	Next
8	Vector Encoder	$1\times 1\times c_{i}$	Head/Channel-Wise Multiplication

^1^ Here, $w_{i},h_{i},c_{i}$ represent three scales of the output size with $c_{1}=256,c_{2}=512,c_{3}=512$; $w_{i}$ and $h_{i}$ depend on the size of the object.

**Table 2:** Two different novel/base split settings on the NWPU.v2 dataset according to [11].

Split		Novel		Base
1	Airplane	Baseball diamond	Tennis court	Rest
2	Basketball	Ground track field	Vehicle	Rest

**Table 3:** Four different novel/base split settings on the DIOR dataset according to [11].

Split			Novel			Base
1	Baseball field	Basketball court	Bridge	Chimney	Ship	Rest
2	Airplane	Airport	Expressway toll station	Harbor	Ground track field	Rest
3	Dam	Golf course	Storage tank	Tennis court	Vehicle	Rest
4	Express service area	Overpass	Stadium	Train station	Windmill	Rest

**Table 4:** The comparison results of our proposed B-FSDet with SOTA few-shot object detectors on the NWPU.v2 dataset. We set K = 3, 5, 10, 20 in our experiments. All data represent the averaged results of three random experiments.

	Methods	Year	3-Shot		5-Shot		10-Shot		20-Shot	
			bAP	nAP	bAP	nAP	bAP	nAP	bAP	nAP
Split 1	TFA [37]	ICML2020	**89.35**	8.80	**89.60**	9.49	**89.95**	9.26	89.62	10.83
	P-CNN [32]	TGRS2021	82.84	41.80	82.89	49.17	83.05	63.29	83.59	66.83
	FsDetView [10]	TPAMI2022	87.68	24.56	87.77	29.55	87.75	31.77	87.83	32.73
	SAGS&TFS [33] ^1^	AEORS2022	—	51.00	—	**66.00**	—	**72.00**	—	—
	G-FSDet [35]	ISPRS2023	**89.11**	49.05	88.37	56.10	88.40	71.82	**89.73**	75.41
	SAE-FSDet [15] ^2^	TGRS2024	—	**57.96**	—	59.40	—	71.02	—	**85.08**
	B-FSDet (ours)	—	88.54	**75.27**	**90.98**	**87.83**	**92.23**	**90.90**	**93.85**	**90.12**
Split 2	TFA [37]	ICML2020	**90.14**	11.14	**91.19**	12.46	**90.79**	11.35	90.37	11.56
	P-CNN [32]	TGRS2021	81.03	39.32	81.18	46.10	80.93	55.90	81.21	58.37
	FsDetView [10]	TPAMI2022	88.11	39.01	89.34	40.31	89.34	45.09	89.31	46.28
	G-FSDet [35]	ISPRS2023	**89.99**	**50.09**	**90.52**	**58.75**	89.23	**67.00**	**90.61**	**75.86**
	B-FSDet (ours)	—	89.00	**61.46**	89.96	**68.86**	**91.76**	**81.64**	**92.58**	**87.64**

^1^ The method SAGS&TFS [33] only considers nAP evaluation and provides data only for split 1 of the NWPU.v2 dataset. ^2^ The method SAE-FSDet [15] shares the same characteristics as mentioned above.

**Table 5:** The accuracy results for each class on the NWPU.v2 dataset. All the results are obtained through three random experiments. The highest and lowest values are highlighted in bold.

	Class/Shot	3-Shot	5-Shot	10-Shot	20-Shot
Base	Airplane	**99.50**	**99.50**	**99.50**	**99.50**
	Baseball diamond	96.93	97.43	96.20	96.80
	Bridge	63.63	63.80	72.40	81.23
	Harbor	86.73	85.97	90.90	87.87
	Ship	90.27	93.33	96.17	95.17
	Storage tank	93.23	94.70	95.97	93.60
	Tennis court	92.73	94.97	91.20	93.90
Novel	Basketball	63.57	71.73	79.80	88.63
	Ground track field	81.77	92.00	96.17	98.73
	Vehicle	**39.03**	**42.83**	**68.97**	**75.57**

**Table 6:** The comparison results of our proposed B-FSDet with SOTA few-shot object detectors on the DIOR dataset. We set K = 3, 5, 10, 20 in our experiments. All data represent the averaged results of three random experiments.

	Methods	Year	3-Shot		5-Shot		10-Shot		20-Shot	
			bAP	nAP	bAP	nAP	bAP	nAP	bAP	nAP
Split 1	TFA [37]	ICML2020	**70.32**	11.35	**70.51**	11.57	**70.52**	15.37	**71.07**	17.96
	P-CNN [32]	TGRS2021	47.00	18.00	48.40	22.80	50.90	27.60	52.20	29.60
	FsDetView [10]	TPAMI2022	59.54	13.19	58.58	14.29	59.64	18.02	62.69	18.01
	SAGS&TFS [33] ^1^	AEORS2022	—	**29.30**	—	31.60	—	31.60	—	40.20
	G-FSDet [35]	ISPRS2023	68.94	27.57	69.52	30.52	69.03	**37.64**	69.80	39.83
	SAE-FSDet [15] ^2^	TGRS2024	—	28.80	—	**32.40**	—	37.09	—	**42.46**
	B-FSDet (ours)	—	**75.95**	**32.02**	**76.33**	**40.49**	**75.65**	**44.00**	**75.53**	**45.90**
Split 2	TFA [37]	ICML2020	**70.75**	5.77	**70.79**	8.19	**69.63**	8.71	**70.02**	12.18
	P-CNN [32]	TGRS2021	48.90	**14.50**	49.10	14.90	52.50	18.90	51.60	22.80
	FsDetView [10]	TPAMI2022	58.88	10.83	60.31	9.63	61.16	13.57	61.16	14.76
	SAGS&TFS [33]	AEORS2022	—	12.60	—	15.50	—	15.50	—	**23.80**
	G-FSDet [35]	ISPRS2023	69.20	14.13	69.25	**15.84**	68.71	**20.70**	68.18	22.69
	SAE-FSDet [15]	TGRS2024	—	13.99	—	15.65	—	17.41	—	21.34
	B-FSDet (ours)	—	**72.19**	**21.54**	**73.06**	**29.76**	**73.42**	**40.67**	**73.14**	**51.51**
Split 3	TFA [37]	ICML2020	**71.95**	8.36	**71.64**	10.13	**72.56**	10.75	**73.13**	17.99
	P-CNN [32]	TGRS2021	49.50	16.50	49.90	18.80	52.10	23.30	53.10	28.80
	FsDetView [10]	TPAMI2022	61.00	7.49	61.33	12.61	61.94	11.49	65.17	17.02
	SAGS&TFS [33]	AEORS2022	—	**20.90**	—	**24.80**	—	24.80	—	**36.10**
	G-FSDet [35]	ISPRS2023	71.10	16.03	70.18	23.25	71.08	26.24	71.26	32.05
	SAE-FSDet [15]	TGRS2024	—	16.74	—	19.07	—	**28.44**	—	29.88
	B-FSDet (ours)	—	**74.03**	**31.96**	**73.37**	**37.88**	**74.00**	**45.21**	**74.18**	**52.55**
Split 4	TFA [37]	ICML2020	68.57	10.42	**68.85**	14.29	**68.58**	14.35	**68.86**	12.01
	P-CNN [32]	TGRS2021	49.80	15.20	49.90	17.50	51.70	18.90	52.30	25.70
	FsDetView [10]	TPAMI2022	58.90	14.28	58.97	15.95	60.37	15.37	60.89	16.96
	SAGS&TFS [33]	AEORS2022	—	**17.50**	—	19.70	—	19.70	—	27.70
	G-FSDet [35]	ISPRS2023	**69.01**	16.74	67.96	**21.03**	68.55	**25.84**	67.73	**31.78**
	SAE-FSDet [15]	TGRS2024	—	**17.27**	—	20.48	—	22.69	—	26.75
	B-FSDet (ours)	—	**74.18**	17.01	**73.47**	**26.14**	**73.72**	**36.08**	**72.90**	**44.62**

^1^ The method SAGS&TFS [33] only considers nAP evaluation and only provides nAP of the DIOR dataset. ^2^ The method SAE-FSDet [15] shares the same characteristics as mentioned above.

**Table 7:** The accuracy results for each class on the DIOR dataset. All the results are obtained through three random experiments. The highest and lowest values are highlighted in bold.

	Class/Shot	3-Shot	5-Shot	10-Shot	20-Shot
Base	Airplane	86.63	83.80	85.67	84.93
	Airport	77.00	78.80	81.20	84.00
	Expressway toll station	77.93	75.57	78.37	78.43
	Harbor	58.23	52.23	57.20	56.43
	Ground track field	78.30	76.30	78.40	80.97
	Expressway service area	**90.97**	89.30	**89.33**	85.57
	Overpass	56.67	57.80	59.33	60.10
	Stadium	70.23	72.50	75.50	72.43
	Train station	65.13	60.77	59.73	64.50
	Windmill	88.53	87.00	87.60	**90.23**
	Baseball field	81.40	80.53	79.27	79.90
	Basketball court	89.60	**89.80**	88.83	89.80
	Bridge	37.97	41.27	44.23	45.37
	Chimney	82.83	81.60	83.33	83.00
	Ship	69.07	73.27	62.07	57.10
Novel	Dam	**10.42**	**8.65**	**20.37**	**34.07**
	Golf course	24.30	36.63	54.40	63.03
	Storage tank	37.00	49.77	46.67	49.63
	Tennis court	72.77	76.20	77.30	81.07
	Vehicle	15.33	18.17	27.30	34.93

**Table 8:** Results of ablation experiments conducted on the NWPU.v2 dataset. All the results are obtained through averaging three independent experimental trials.

Baseline (YOLOv9)	S-Meta ^1^	DCS	ICDSL	3-Shot		5-Shot		10-Shot		20-Shot	
				bAP	nAP	bAP	nAP	bAP	nAP	bAP	nAP
✓				84.83	56.89	80.40	62.73	77.08	76.50	80.44	83.01
✓	✓			85.24	52.67	83.63	67.07	87.09	80.69	87.49	83.69
✓	✓	✓		**90.93**	**71.67**	**91.91**	**81.92**	**91.80**	**86.06**	**92.18**	**89.71**
✓	✓	✓	✓	**88.54**	**75.27**	**90.98**	**87.83**	**92.23**	**90.90**	**93.85**	**90.12**

^1^ The adoption of S-Meta here implies that the baseline is transformed into a meta-learning-based few-shot object detector using SFEM and SFPM.

**Table 9:** Results of ablation experiments of inference speed (SFPM) conducted on the NWPU.v2 dataset. The inference time data were obtained with the GPU preheated and no other operations such as saving visualization results performed.

Baseline (YOLOv9)	S-Meta ^1^	SFPM	Inference Time	3-Shot		5-Shot		10-Shot		20-Shot	
				bAP	nAP	bAP	nAP	bAP	nAP	bAP	nAP
✓			**9.1 ms**	84.83	56.89	80.40	62.73	77.08	76.50	80.44	83.01
✓	✓		33.5 ms	**88.43**	**76.82**	**91.10**	**86.06**	**93.36**	**91.77**	**93.31**	**92.83**
✓	✓	✓	**9.5 ms**	**88.54**	**75.27**	**90.98**	**87.83**	**92.23**	**90.90**	**93.85**	**90.12**

^1^ Here, S-Meta represents the exclusion of the SFPM method and it employs the prediction method from the previous meta-learning-based detectors [11,12]. Furthermore, the DCS and ICDSL methods have also been incorporated.

**Table 10:** The experimental results of inference speed and resource consumption of B-FSDet across different devices. All devices utilized in the experiments are NVIDIA-produced GPUs.

GeForce RTX 1080 Ti		GeForce RTX 2080 Ti		GeForce RTX 3090		GeForce RTX 4090D	
Speed	Consumption ^1^	Speed	Consumption	Speed	Consumption	Speed	Consumption
31.90 fps	9.87 M	105.26 fps	8.21 M	107.53 fps	8.21 M	166.67 fps	8.21 M

^1^ “Consumption“ refers to GPU memory usage under the aforementioned experimental settings.

**Table 11:** Results of different DCS implementations on the NWPU.v2 dataset. All data represent the averaged results of three random experiments.

DCS	3-Shot		5-Shot		10-Shot		20-Shot	
	bAP	nAP	bAP	nAP	bAP	nAP	bAP	nAP
BP	**90.51**	**75.14**	**90.53**	86.14	90.76	86.69	92.99	89.46
GM	**89.22**	75.07	89.90	**89.57**	**91.61**	**89.47**	**93.34**	**92.67**
WGN	88.54	**75.27**	**90.98**	**87.83**	**92.23**	**90.90**	**93.85**	**90.12**

## Data Availability

The data used in this study were derived from the following resources available in the public domain: [https://doi.org/10.1109/TGRS.2017.2778300 and https://doi.org/10.1016/j.isprsjprs.2019.11.023].
